# True Giant Posterior Tibial Artery Aneurysm

**DOI:** 10.1155/2012/695250

**Published:** 2012-12-10

**Authors:** Alessandro Robaldo, Giacomo Di Iasio, Gabriele Testi, Patrizio Colotto

**Affiliations:** Vascular and Endovascular Surgery Unit, Imperia Hospital, Via Sant'Agata 57, 18100 Imperia, Italy

## Abstract

We report an unusual case of true atherosclerotic posterior tibial artery (PTA) aneurysm without any apparent causative history. To our knowledge, in the English Literature only seven previously cases of true PTA aneurysms are reported. Due to its location, this lesion may require surgical intervention and removal. The presentation, the diagnostic evaluation, and the surgical management of the aneurysm are discussed.

## 1. Clinical History 

**Figure 1:**
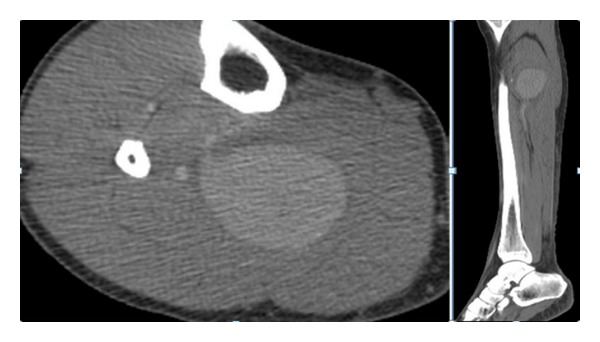
The giant posterior tibial artery aneurysm was diagnosed by angio-CT scan.

**Figure 2:**
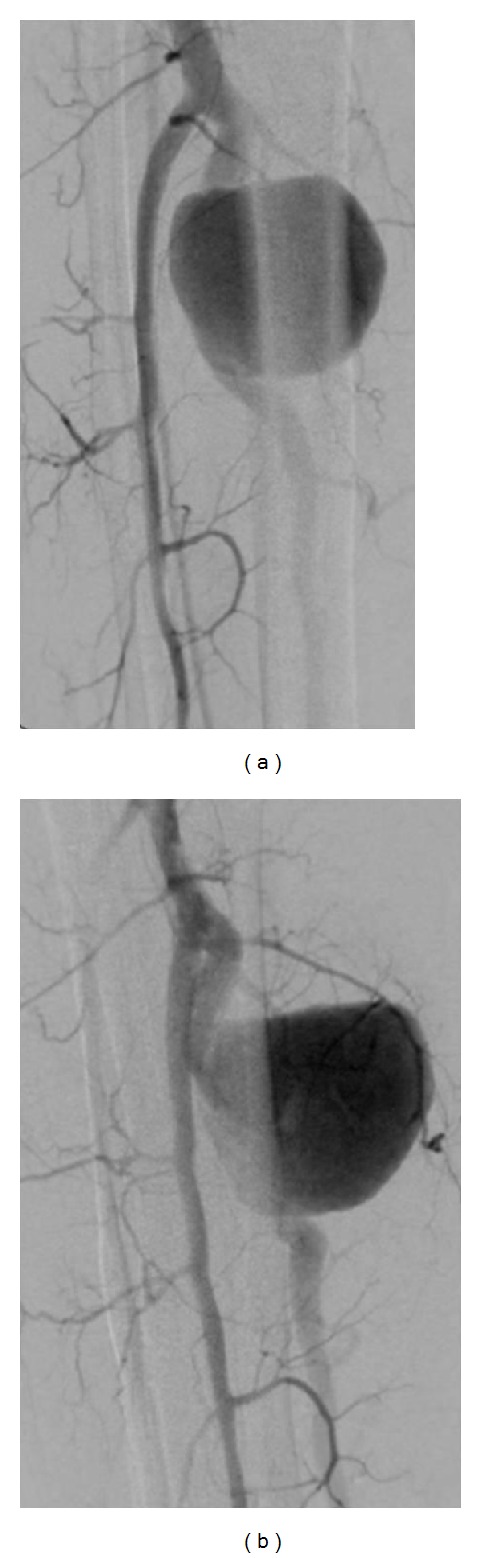
Posterior tibial artery aneurysm detected by angiography scan.

**Figure 3:**
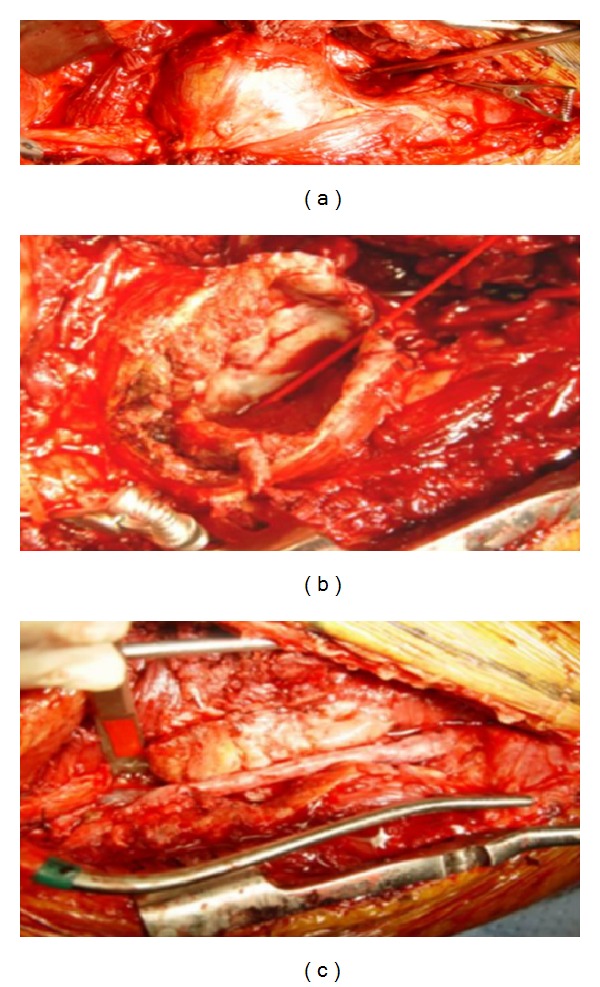
Intraoperative image of the true giant posterior tibial artery aneurysm. On the left, the aneurysm is opened with a large thrombus. On the right, resection and interposition of a reversed saphenous vein segment.

We report the diagnostic and therapeutic images of a true atherosclerotic posterior tibial artery (PTA) aneurysm without any apparent causative history. A 52-year-old male patient presented himself with a pulsatile painful mass of the right lower leg. The patient's medical history was completely negative and the clinical condition was good. No fever was detected. The CPR and blood culture results were negative. The duplex scan (DS) revealed a giant PTA aneurysm (about 50 mm in diameter). The angio-CT scan detected the patent saccular aneurysm at the proximal segment of the posterior tibial artery with a transversal maximum diameter of 53 mm and concentric mural thrombus. No abnormalities of the bones, namely, sharp spikes that could damage the tibial artery, were recognized. No other aneurysm sites were detected (Figure 1). A selective angiography scan (AS) confirmed the diagnosis (Figure 1) and showed the patency of the tibial and peroneal arteries (Figure 2). Under spinal anesthesia, through a medial surgical approach, the aneurysm was repaired successfully by resection and interposition of a reversed saphenous vein segment. The aneurysm wall (Figure 3), sent for the histologic examination, showed a partially thrombosed true atherosclerotic aneurysm without signs of connective tissue disorders, arteritis, vasculitis, or infection. The patient was discharged in good general condition with a complete pain relief, regular pulse, and life-long mono-antiplatelet treatment (acetylsalicylic acid 300 mg). At the 12-month followup, no foot or digital ischemia, complain of paresthesias, pain, discomfort, or walking limitation has been observed. Duplex scan showed a good patency in the absence of stenosis, pseudoaneurysms, or recurrent aneurysms. 

## 2. Comments 

The majority of the PTA aneurysms are not frequent and usually they are secondary to trauma, infection, or iatrogenic injury. In the English Literature, few cases of true atherosclerotic PTA aneurysm had previously been reported [1–6]. The indications for treating these lesions are debated, but usually symptomatic aneurysms such as asymptomatic large aneurysms or those with laminated thrombus should be treated [2, 6]. The choice of treatment (surgical/endovascular) depends on the location, shape, and size of the aneurysm, as well as the patient's general condition [3, 5]. The surgical repair is typically achieved with autogenous vein bypass grafting; artery direct suture is described with good success for focal lesions. When there is an adequate collateral circulation, artery ligation can be a second option [1, 5, 6]. The endovascular treatment (coil embolization, thrombin glue injection, covered stent) is a good alternative to surgery and is preferred in case with high-risk patients [3]. In this case, considering the large dimension of the aneurysm, we preferred surgical option to excise the aneurysm sac which determined a compartment pain in the leg and to prevent endovascular embolization in the tibial vessels. Treatment should be individualized time by time by evaluating patients' comorbidities and aneurysm conformation.
